# Structural and functional insights into esterase-mediated macrolide resistance

**DOI:** 10.1038/s41467-021-22016-3

**Published:** 2021-03-19

**Authors:** Michał Zieliński, Jaeok Park, Barry Sleno, Albert M. Berghuis

**Affiliations:** 1 Department of Biochemistry, McGill University, Montréal, QC Canada; 2 Centre de Recherche en Biologie Structurale, McGill University, Montréal, QC Canada; 3 Department of Microbiology and Immunology, McGill University, Montréal, QC Canada; 4 Present Address: Department of Biochemistry, Memorial University of Newfoundland, St John’s, Newfoundland and Labrador Canada

**Keywords:** Hydrolases, Proteins, X-ray crystallography, Antibiotics

## Abstract

Macrolides are a class of antibiotics widely used in both medicine and agriculture. Unsurprisingly, as a consequence of their exensive usage a plethora of resistance mechanisms have been encountered in pathogenic bacteria. One of these resistance mechanisms entails the enzymatic cleavage of the macrolides’ macrolactone ring by erythromycin esterases (Eres). The most frequently identified Ere enzyme is EreA, which confers resistance to the majority of clinically used macrolides. Despite the role Eres play in macrolide resistance, research into this family enzymes has been sparse. Here, we report the first three-dimensional structures of an erythromycin esterase, EreC. EreC is an extremely close homologue of EreA, displaying more than 90% sequence identity. Two structures of this enzyme, in conjunction with in silico flexible docking studies and previously reported mutagenesis data allowed for the proposal of a detailed catalytic mechanism for the Ere family of enzymes, labeling them as metal-independent hydrolases. Also presented are substrate spectrum assays for different members of the Ere family. The results from these assays together with an examination of residue conservation for the macrolide binding site in Eres, suggests two distinct active site archetypes within the Ere enzyme family.

## Abstract (web summary)

Erythromycin esterases (Eres) cleave the macrolactone ring of macrolides, a class of widely used antibiotics. Structures of EreC, in silico flexible docking studies and previous mutagenesis data lead to the proposal of a detailed catalytic mechanism for the Ere family of enzymes.

## Introduction

Since their discovery in the 1950s, macrolide antibiotics have found application in clinical and agricultural settings for the treatment of bacterial infections. Most notably, in clinical settings they have been extensively prescribed for ear, nose, throat, chest, and skin infections, as well as sexually transmitted infections such as chlamydia^1^. Their canonical chemical structure is very modular, with three distinct components: the macrolactone ring of varying sizes, sugar on the 3-position, and an aminosugar on the 5-position (Supplementary Fig. 1). Next-generation macrolides, known as ketolides, lack a sugar at the 3-position, replaced instead by a ketone moiety. This modification has allowed for a decrease in macrolide susceptibility to some resistance factors, allowing them to retain their antibiotic activity. Due to the preserved core structure in macrolides, the mode by which they exert their effect is largely conserved. The vast majority of these antibiotics bind in the peptide exit tunnel of the 50S ribosomal subunit, blocking the lumen, thereby prohibiting the elongation of the growing polypeptide^2–6^. Depending on the macrolide, this mechanism of action can be bactericidal or bacteriostatic^7^.

Bacteria have developed a plethora of ways to circumvent the antibiotic properties of macrolides. The most studied methods include decreasing intracellular concentration via the use of efflux pumps^8,9^, ribosome modification^10,11^, ribosome protection^12,13^, and macrolide phosphotransferase (MPH) mediated modification^14,15^. A much-overlooked mechanism by which bacteria can detoxify macrolides is macrolactone ring cleavage catalyzed by erythromycin esterases (Ere)^14^.

The first Ere enzyme (EreA) was identified in 1984, from a hospital-derived strain of *Escherichia coli* resistant to erythromycin^16^. EreB was discovered a year later in *E. coli*^17^. Decades later, EreC was found in multidrug-resistant *Klebsiella pneumoniae*^18^, and EreD in a duck pathogen, *Riemerella anatipestifer*^19^. The prevalence of erythromycin esterases in clinically relevant strains has increased as the genes coding for Ere family of proteins can be found on plasmids for all members of this family, except EreD^14^. Notably, within the Ere family, EreA appears to be the enzyme most often identified in clinical clinical strains such as *Pseudomonas spp*.^20^, non-typhoidal *Salmonella enterica*^21^*, Vibrio cholera*^22^, MRSA^23^, and *Klebsiella oxytoca*^24^. Intriguingly, EreB is frequently detected in environmental isolates^14^. EreC, due to it being a recent addition to the Ere family, has largely stayed under the radar in macrolide resistance surveillance. However, in addition to multidrug-resistant Klebsiella pneumoniae^18^, a search of EreC in sequence data banks reveals its presence in *Enterobacteriaceae* and *E. coli*. For more comprehensive discussion on the spread of erythromycin esterases we would like to direct the reader to the review by Golkar et al.^14^. With respect to their sequence conservation, Eres represent a diverse family of proteins. EreA and EreC are extremely closely related, displaying a 90.2% sequence identity and 94.3% similarity. EreB and EreD are relatively closely related with a 44.7% sequence identity; however, they are distantly related to EreA and EreC, sharing no more than 25.4% sequence identity^14^.

Initial work into the elucidation of the enzymatic mechanism of erythromycin esterases began with the publication by Morar et al.^25^ on EreA and EreB. Morar and colleagues made several key observations: It was demonstrated that H50 (EreC numbering) would be the most fitting candidate to play the role of a catalytic base, since it is a highly conserved residue, which, upon its mutation, causes a 1000-fold reduction in erythromycin degradation. E78A mutation was the only one to completely abolish all catalytic activity, while H289A reduced K_cat_ 1000-fold. However, it was later speculated that H289 and E78 are responsible for substrate specificity rather than catalysis. It was also reported that the mutation E47A reduces the catalytic rate of the enzyme, as the loss of glutamic acid would result in greater rotameric freedom of H50. This study, however, lacked three-dimensional structures of any member of the Ere family, preventing it from suggesting a possible catalytic mechanism for this class of antibiotic resistance enzymes.

Here, we determine the three-dimensional, X-ray crystal structure of EreC in two different conformations. In addition, docking simulations to explore the details of macrolide binding are performed. Combined these studies provide detailed insights into the catalytic mechanism. Furthermore, substrate spectrum analyses of EreC and other members of the Ere family are presented, which combined with the structural data provide information on the basis for broad substrate spectrum for this class of antibiotic resistance enzymes.

## Results

### Structure characteristics

**Fig. 1:**
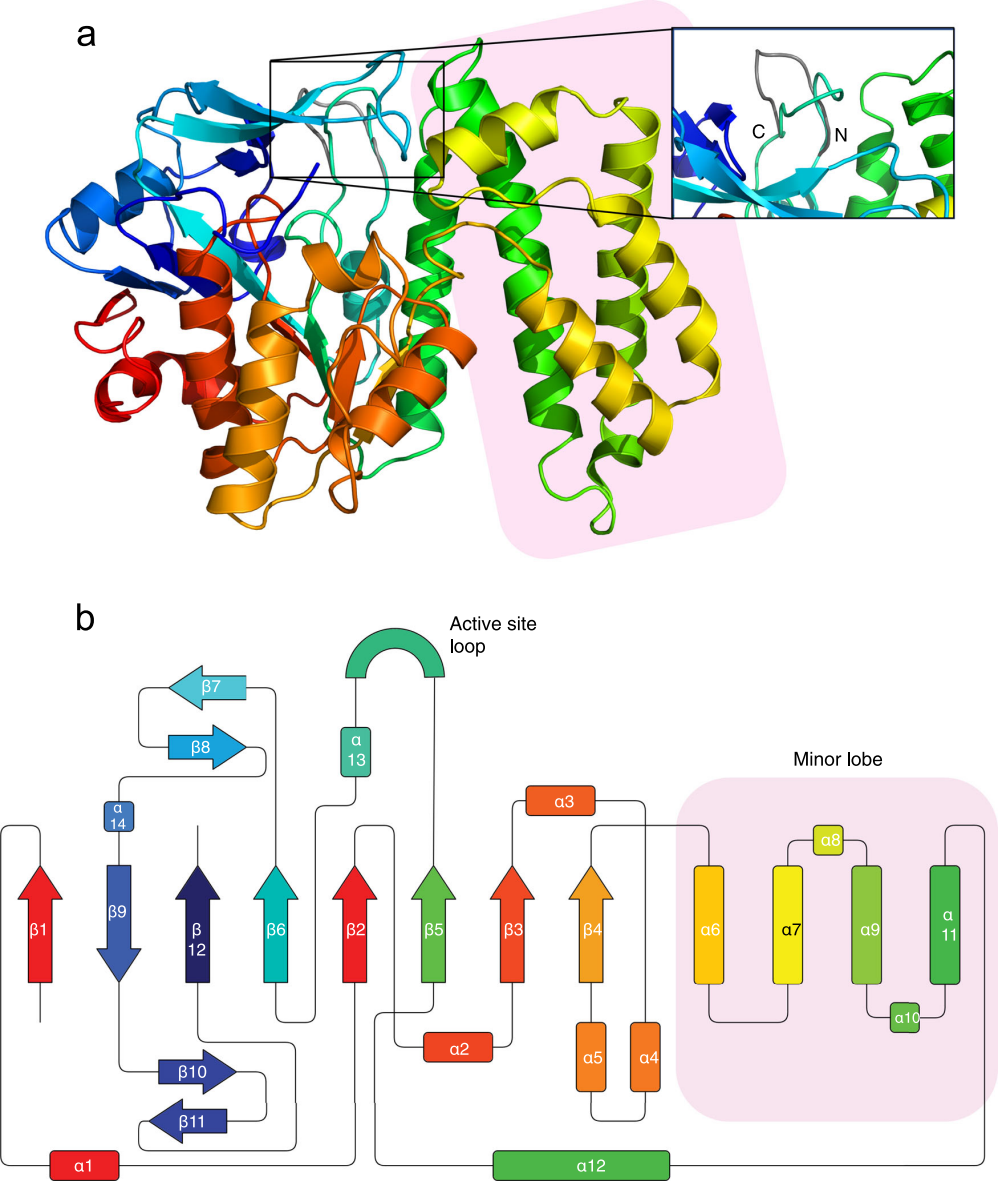
Three-dimensional structure and topological diagram of EreC. Overall structure of EreC^Closed^ in a cartoon representation with a rainbow color scheme (red at the N-terminus to blue at the C-terminus). The loop in EreC^Open^ is presented in gray as a composite structure on top of EreC^Closed^. An optimized view of the loop conformations is shown in the frame. **b** Topological diagram of the polypeptide fold of EreC with β-sheets indicated by arrows, α-helices by rectangles, and the active-site loop by a semi-circle. Color code follows the one from (**a**).

We determined the three-dimensional structure of EreC H289N in two different crystal forms. Crystal form I, determined to a resolution of 2.0 Å, has one molecule per asymmetric unit, and crystal form II was resolved to 2.4 Å and has two molecules of EreC per asymmetric unit. Both crystal forms show that the protein can be divided into two parts, a major and a minor lobe (Fig. 1). The major lobe makes up the greater portion of the protein and is composed of 8 β-strands (β1–6, β9, and β12) forming a continuous sheet, surrounded by α-helices and loops. The remaining portion of the protein makes up the minor lobe and contains a four-helix bundle motif (α6, α7, α9, and α11) (Fig. 1). This particular fold is not novel as it has been previously seen in five other structures. BcR135 (used as the search model for MR) and BcR136 from *Bacillus cereus*, to ChaN from *Campylobacter jejuni*^26^, HopBA1 from *Pseudomonas syringae*^27^, and PMT from *Pasteurella multocida*^28^ (Supplementary Fig. 2). However, only BcR135 and BcR136 possess both the major and minor lobe, the remaining three proteins lack the minor lobe. The biological function of these proteins is highly diverse, with BcR135 and BcR136 being implicated in succinoglycan biosynthesis, ChaN in heme transport, HopBA1 being a type III effector protein, and PMT a toxin. Moreover, esterase activity is only shown or assumed for BcR135 and BcR136^25^, the activity for the other proteins is unknown or radically different, e.g., ChaN binds heme^26,27^. This is not unexpected as it has been noted for other antibiotic resistance enzyme that they exploit folds used in different tasks^29^.

Most of the EreC structure is effectively identical in the two crystal forms, as is reflected in pairwise RMSD values of 0.25–0.41 Å between the three crystallographically independent EreC molecules. However, there is a notable difference in a loop between residues 295 and 303 (Fig. 1). In crystal form I, this loop is folded over the rest of the enzyme, and we have designated this the closed configuration (EreC^Closed^). In crystal form II, the loop is directed away from the rest of the enzyme, and we have called this the open configuration (EreC^Open^). Not unexpectantly, electron density corresponding to erythromycin could not be explicitly identified in either of the two structures. Therefore, the two structures of EreC are henceforth described as apo-structures.

### Modeled interaction between EreC and erythromycin

Despite the presence of erythromycin during crystallization, the structures did not reveal how the macrolide binds to EreC. To examine the binding of erythromycin to EreC, flexible docking (Dock v6.9) was used to build a model of the EreC^Closed^-erythromycin complex. The pocket in which the docking experiments were conducted was guided by work done by Morar and colleagues, where catalytically crucial amino acids (H289, H50, E78, and E47; EreC numbering) were identified for EreB^25^. As EreC is an esterase, it requires the use of water to perform its reaction; as such, the presence of water would be useful in our docking experiment. We selected a water molecule present in the close vicinity of catalytic residues (H50, E78, and H289) that is conserved in both the open and closed conformations of the enzyme, strongly suggesting it is the second substrate in the hydrolysis reaction.

The rationale behind utilizing the closed conformation of EreC for the docking experiment was because of the higher resolution structure, providing greater confidence in the position of the amino acid conformers. It was realized that the closed position of the flexible loop could potentially interfere with the placement of macrolide. However, it was found that the closed conformation of the loop was actually compatible with macrolide binding, and it suggests that the closing of the loop can be important for catalysis.

**Fig. 2:**
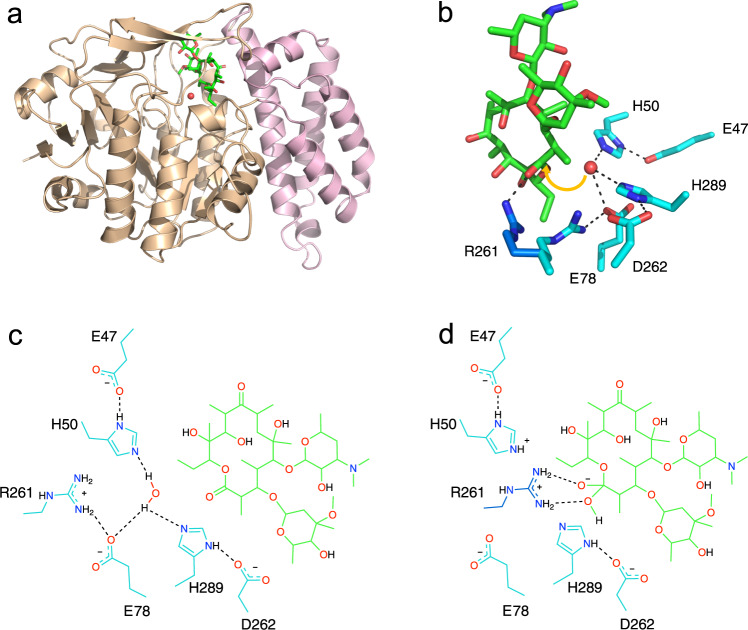
Predicted binding mode of erythromycin. Erythromycin modeled into the WT EreC^Closed^ model showing catalytic amino acids and the interactions between the residues and the substrate. **a** Modeled structure of EreC^Closed^ with erythromycin and water. The major lobe is displayed in light brown and the minor lobe in light pink. **b** A three-dimensional representation where protein residues are colored in teal, modeled alternate side-chain conformation of R261 in dark blue, erythromycin in green, and water molecule in red. The arrow indicating where the water molecule will attack erythromycin is shown in yellow. **c** Two-dimensional representation displaying the residues implied in the catalytic activity together with erythromycin. The color scheme of carbon atoms is the same as in (**b**)**. d** Modification of (**c**) showing modeled alternate side-chain conformation of R261, and how it can interact with the transition state.

In total, 51 conformers were generated, with a grid score ranging between −84 and −9. A conformer with a grid energy score of −82.5 was selected due to its favorable positioning in the active pocket, the proximity of the erythromycin’s ester bond to the active site residues and the closeness of the postulated hydrolysing water to the ester. Arriving at the model for macrolide binding to EreC is shown in Fig. 2.

Upon examining the docked structure, we were able to see that erythromycin sits snugly in the active site of EreC^Closed^ with the interfacial surface area of 633 Å^2^, as calculated by PDBePISA. The hydrophobic face of erythromycin is forming interactions with hydrophobic patches in the binding site, and some electrostatic interactions and hydrogen bonds are also predicted to occur (Supplementary Fig. 3). Because of the criteria used for identifying the EreC-erythromycin complex model, the putative hydrolysing water molecule is adjacent to the macrolide’s ester bond. In support of this model, the water molecule is suitably located at the *si-*face of the ester moiety, and 3.8 Å from the carbonyl carbon atom for nucleophilic attack. Also, the R261 side chain is able to adopt an alternate conformer such that its guanidino group forms favorable hydrogen bond and charge interactions with erythromycin’s two oxygen atoms in a tetrahedral transition state (Fig. 2).

### Enzymatic assays

LC-MS was used to determine whether EreB, EreC and EreD were capable of performing the macrolactone ring-opening reaction on 13 diverse macrolides, of which several are clinically relevant (Supplementary Fig. 4). EreA was not included in the panel of antibiotic resistance enzymes tested as it is virtually identical in protein sequence to EreC (see Discussion). Out of the three enzymes tested, EreC has the broadest substrate specificity, including 14-membered macrolides and ketolides, 15-membered azithromycin and two 16-membered macrolides, josamycin and midecamycin. EreB and EreD displayed nearly identical substrate specificities, where degradation of all tested 14-membered non-ketolide macrolides and the 15-membered azithromicycin was achieved. It was determined that EreB and EreD are unable to use any of the tested 16-membered species as substrates. None of the three enzymes can catalyze the degradation of 15-membered tulathromycin and 16-membered spiramycin (see Table 1 and Supplementary Fig. 5).

**Table 1:** Activity of erythromycin esterases against a panel of macrolides.

Ring size	Macrolide	EreC	EreB	EreD
14	Clarithromycin	S	S	S
	Erythromycin	S	S	S
	Oleandomycin	S	S	S
	Pikromycin	S	S	S
	Roxithromycin	S	S	S
	Flurithromycin	S	S	S
	Solithromycin^k^	S	PS	NS
	Telithromycin^k^	NS	NS	NS
15	Azithromycin	S	S	S
	Tulathromycin	NS	NS	NS
16	Spiramycin	NS	NS	NS
	Josamycin	PS	NS	NS
	Midecamycin	PS	NS	NS

Substrates are classified as substrate (S) if >85% of macrolide was degraded, poor substrate (PS) for 30–85% degraded, and non-substrate (NS) for <30% degraded.

^k^-ketolide antibiotic.

### Modeled interaction between EreC and other macrolides

**Fig. 3:**
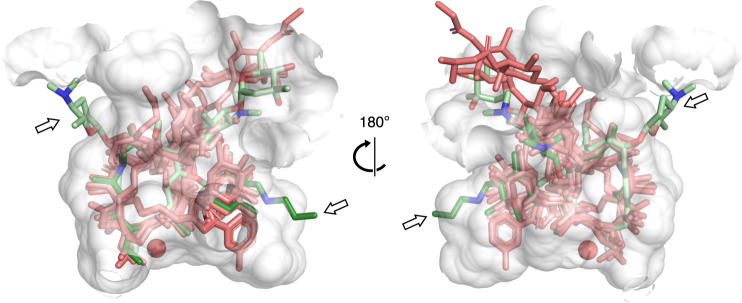
Macrolide substrates and non-substrates in the active site of EreC. Various macrolides modeled into the active site of EreC based on the conformation of erythromycin. The gray surface represents the active site of EreC^Closed^. Macrolides that are a substrate of EreC are colored in pink, spiramycin is colored in light green, and tulathromycin is colored in dark green. The catalytic water is represented as a red sphere. The arrows indicate the position of protein-macrolide steric clashes.

The erythromycin-docked structure of EreC was used as a template to model other macrolides in the binding pocket of EreC^Closed^. Modeling of the macrolides was performed by super-positioning them so that the orientation of the ester bond was identical amongst all the macrolactone rings. Two macrolides proved difficult to model in the active site due to steric clash. For spiramycin, this is due to its forsamine group at position-9 is clashing with the loop between β7 and β8. For tulathromycin, it is due to its *N*-methylpropylamine group on the cladinose sugar clashing with α12 (Fig. 3). The other macrolides overlaid well within the active site without steric clashes.

## Discussion

**Fig. 4:**
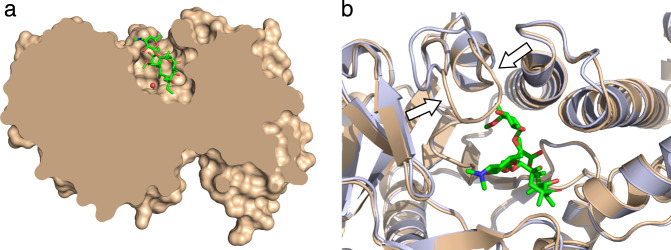
Cross-section of EreC with emphasis on the flexible loop. **a** A cut-through section of EreC^Closed^ with docked erythromycin shows the depth of the catalytic pocket and the protein-ligand interface. **b** View rotated 90° along the horizontal axis, with EreC^Closed^ colored in light brown and EreC^Open^ colored in light blue. The active site loop is in two distinct conformation (arrows), which can cover the top of the active site and act as a lid.

Erythromycin esterases have been a largely neglected family of antibiotic resistance enzymes, with few studies tackling the elucidation of their mechanism of action. The closest structural model available is of distantly related enzymes that are capable of esterase activity on molecules, such as *p*-nitrophenyl butyrate, thought to be involved in succinoglycan biosynthesis in *Bacillus cereus* (BcR135 and BcR136)^25^. Here we showcase the first structure of an erythromycin esterase family enzyme capable of hydrolyzing macrolides (EreC). The structural determination of this enzyme bound to a macrolide substrate has proven to be a significant challenge. Instead, we have used molecular docking to elucidate how EreC may interact with erythromycin. We have found erythromycin to be deeply buried in a pocket (Fig. 4). The macrolide binding site itself displays similar characteristics to those belonging to a MPHs (MPH(2′)-I MPH(2′)-II) and the ribosome^15^. All of the aforementioned binding sites have a hydrophobic and a hydrophilic face, which allows for complimentary interactions with the corresponding faces of the macrolide. The conformation of the modeled macrolide, though left flexible during docking, is essentially identical to that found in MPHs and the ribosome (e.g., RMSD of 0.17 Å and 0.53 Å with MPH(2′)-I and the ribosome, respectively^15^). However, it is important to note that this structure has been generated via docking, and an X-ray crystal structure of EreC in complex with macrolide would be needed to describe the binding in greater detail.

By comparing EreC^Open^ and EreC^Closed^, in conjunction with the docking results, we were able to identify a loop rich in glycine and proline, which is capable of closing over the active site (Fig. 4). We hypothesize that this flexible loop closes upon substrate binding and returns to the open conformation after reaction completion, to ready the active site for another cycle of catalysis.

**Fig. 5:**
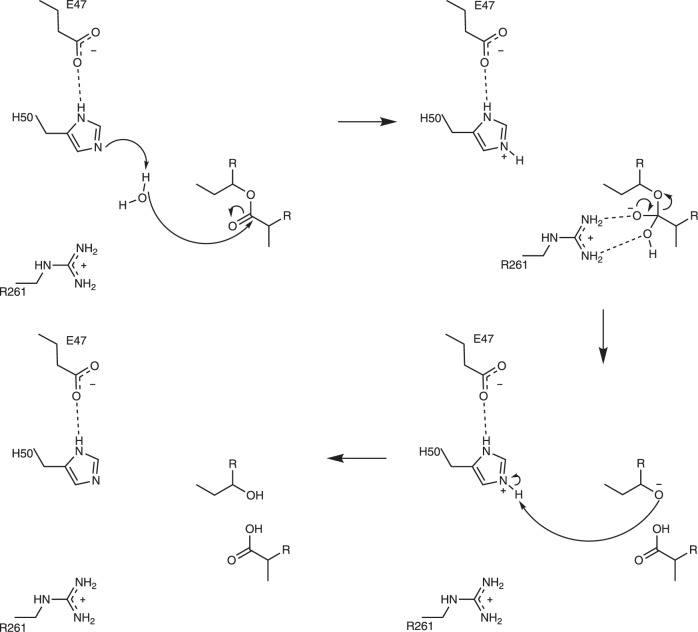
Catalytic mechanism of EreC. Proposed enzymatic reaction mechanism employed by EreC, displaying a cropped molecule of erythromycin and amino acids directly implied in catalysis.

Using our model of the EreC^Closed^-erythromycin complex obtained by docking and mutagenesis data reported by Morar et al., we can provide mechanistic insights into Ere mediated catalysis. The solved three-dimensional structures combined with previously published data, further supported by our own analysis (Supplementary Fig. 6) allowed us to designate EreC as a metal-independent hydrolase with nucleophilic water (group 3–4) according to the nomenclature proposed by Oh et al.^30^ In analogy to the catalytic mechanism of the carboxylic acid esterase, LigI^31^, we can propose the following catalytic mechanism for EreC. Upon the binding of erythromycin, the flexible loop of EreC closes over the active site, positioning the ester bond within the vicinity of residues H50, E78, and H289. These three residues coordinate the catalytic water, positioning it correctly for a nucleophilic attack. The conformation of the H50 side chain is further stabilized via interaction with E47, decreasing its pKa and increasing the histidine’s ability to act as a catalytic base. The reaction starts with deprotonation of the catalytic water by H50, forming the hydroxide ion, which proceeds to attack the partially positive carbon of the ester bond. A repositioning of the R261 sidechain then enables the stabilization of the resulting negative charge present on the transition state intermediate. The deprotonated alcohol on the cleaved erythromycin product then sequesters the proton previously abstracted by H50, resulting in the completion of the reaction and subsequent release of the product (Fig. 5).

**Fig. 6:**
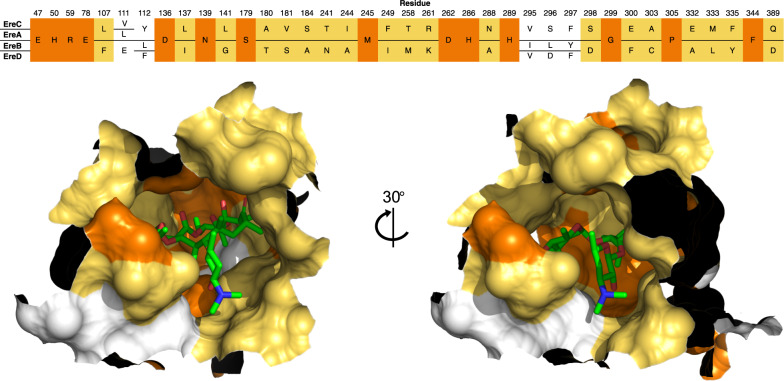
Active site architecture of Eres. Upper panel: MAFFT protein sequence alignment of EreA, EreB, EreC, and EreD. The residues are colored as per sequence identity. The fully conserved residues are in orange, the residues conserved between EreA/C and EreB/D are in yellow, and variable residues are in white. Lower panel: the conservation is shown on protein sequence, as per the coloring in the upper panel, with erythromycin present as a reference.

Next, we determined if the above-mentioned reaction mechanism could be assigned to other members of the Ere family. Following the multiple sequence alignment of protein sequences (in Mafft)^32^ (Fig. 6), it is determined that all of the amino acids implied in EreC-mediated catalysis of erythromycin are also present in all of the other Ere enzymes, with the notable exception of the residue present at position 261. In EreC and EreA, the residue at this position is arginine, while EreB and EreD, it is a lysine. This is not an unexpected find, as both lysine and arginine are basic resides with similar characteristics, allowing for the variation within the protein sequences of distant relatives. In fact, the conservation of the basic nature of amino acids at that position might indicate a strong drive for its conservation and importance in the catalytic mechanism.

The question of how erythromycin esterases can interact with macrolides can be separated into two main considerations. First, how can EreC accommodate a range of different macrolide substrates, and second, what might account for the difference in substrate spectra between different Ere enzymes? Despite macrolides exhibiting variations of chemical groups on their molecular scaffold, EreC can accommodate most of these (Table 1, Fig. 3). An important factor in this might be due to macrolides having a macrolactone ring, which is structurally similar whether we look at 14-, 15- or 16-membered macrolides and contains a hydrophobic moiety that can non-specifically interact with the enzyme. However, due to the chemical nature of tulathromycin and spiramycin, neither of these can bind to the active site, as shown in the model, which is in agreement with our LC-MS assays. This inability to bind is due to the protrusion of different substituents on the macrolides scaffold. Specifically, these substituents include the forsamine group at position 9 on spiramycin and the methylpropylamine at the 4″ of the cladinose on tulathromycin. Despite the sequence divergence of EreB and EreD, the two enzymes present nearly identical spectrum of substrate specificities, showing selectivity towards 14-membered macrolides and 15-membered azithromycin. On the contrary, EreC exhibits a more diverse range, with its ability to inactivate selected 16-membered macrolides (Table 1).

Combining the results of activity assays, multiple sequence alignment, and the structural data of the active site led to the following critical observations. The inspection of the EreC crystal structure active allowed us to identify the residues which make up its active site architecture (Fig. 6). The position of the identified amino acids was then relayed onto a multiple sequence alignment, where patterns between the four enzymes active sites emerged. In total, 38 amino acids line the active site of EreC; 29 contributed by the major lobe and 9 by the minor lobe, with no amino acid implied in catalysis being present on the minor lobe. Out of these 38 residues 14 (37%) are common between all four enzymes. Strikingly, EreB and EreD, despite only having a 44.7% global sequence identity, display a 89% sequence identity and 95% sequence similarity in the active site. Similarly, the active site residues of EreC and EreA exhibit 97% sequence identity and 100% sequence similarity. The only difference in the architecture of this active site is the amino acid 111 (EreC numbering), which is leucine in EreA and valine in EreC. This change is very subtle both in terms of chemistry and the size of the side-chain. As such we expect EreA and EreC to have identical spectra of substrate selectivities. These observations reinforce the results from the enzymatic activity assays, where EreB and EreD exhibit nearly identical spectrum of activity, despite their overall sequence divergence. This has allowed us to propose two separate active site archetypes for the erythromycin esterase family of enzymes. Type I erythromycin esterases, EreA and EreC, and type II erythromycin esterases, EreB and EreD. Despite all the information gathered, we are unable to rationalize the exact differences in the substrate specificity between Type I and Type II Eres. To appropriately address this question, three-dimensional structure of Type I and II Eres with bound macrolides will be necessary.

## Methods

### Expression of erythromycin esterases

EreB (UniProt P05789) was ligated into a pET15b vector (EMD Millipore) and transformed into *E. coli* C43(DE3). Starter culture was prepared by inoculating 5 mL of LB supplemented with 100 mg mL^−1^ ampicillin and growing it overnight at 230RPM and 37 °C. One milliliter of the overnight culture was added into 1 L of LB with 100 mg mL^−1^ ampicillin and grown at 37 °C and 230RPM until optical cell density of 0.6 at *λ* = 600 nm was reached. Protein expression was induced by adding isopropyl β-d-1-thiogalactopyranoside (IPTG) to a final concentration of 1 mM. The temperature was decreased to 20 °C, and the culture was incubated at 230RPM for 17 h. Cells were harvested by centrifugation at 6000 × *g* for 20 min at 4 °C.

EreC (UniProt C7C425) was ligated into a pET15b vector (EMD Millipore) and transformed into *E. coli* C43(DE3). Protein expression was performed by auto-induction in high-density cultures^33^. Overnight starter culture was prepared as described for EreB. Next, 0.25 mL of the overnight culture was added into 12 mL of ZYP-0.8 G with 100 mg mL^−1^ ampicillin and grown at 37 °C and 230RPM for 3 h. Twelve milliliters of the culture in ZYP-0.8 G was then added into 1 L of ZYP-5052 and incubated at 18 °C, 230RPM for 17 h. Cells were harvested by centrifugation at 6000 × *g* for 20 min at 4 °C.

For selenomethionine-labeled EreC (UniProt C7C425) H289N mutant was ligated into a pET15b vector (EMD Millipore) and transformed into *E. coli* B834(DE3). Protein expression was performed by modified auto-induction in high-density cultures, utilizing the incorporation of selenomethionine^34^, at 20 °C, 230RPM for 48 h. Cells were harvested by centrifugation at 6000  × g for 20 min at 4 °C.

EreD (UniProt A0A0H4JQQ7) was ligated into a pET15b vector (EMD Millipore) and transformed into *E. coli* Lobstr BL21(DE3). Protein expression was performed by auto-induction in high-density cultures^33^. Overnight starter culture was prepared as described for EreB. Subsequently, 0.25 mL of the overnight culture was added into 12 mL of ZYP-0.8 G with 100 mg mL^−1^ ampicillin and grown at 37 °C and 230RPM for 3 h. 12 mL of the culture in ZYP-0.8 G was then added into 1 L of ZYP-5052 and incubated at 22 °C, 230RPM for 17 h. Cells were harvested by centrifugation at 6000 × *g* for 20 min at 4 °C.

### Purification of erythromycin esterases

For EreB cells from 1 L of cell growth were resuspended in 30 mL of Ni NTA binding buffer (50 mM HEPES, 150 mM NaCl, 10 mM imidazole pH 7.5). Cells were then lysed by sonication, and cellular debris was removed by centrifugation at 50,000 × *g* for 50 min at 4 °C. Residual cellular debris was removed via filtration through a 0.22 μm syringe-driven filter, and the resulting solution was applied on a prepacked column containing 5 mL of Ni-NTA-Sepharose equilibrated in Ni NTA binding buffer. The protein was eluted using a buffer containing 50 mM HEPES, 150 mM NaCl, 250 mM imidazole pH 7.5 in a step gradient. Fractions containing the protein of interest were identified using SDS-PAGE and pooled. Toyopearl HW-40C column was used to buffer exchange EreB into IEX binding buffer (20 mM Bis-Tris pH 7.0). The protein was again clarified using a 0.22μm syringe-driven filter and applied into an XK 26/40 column containing 100 mL of Q-Sepharose anion exchange resin. The protein was eluted using a gradient 0–100% elution buffer (20 mM Bis-Tris, 1000 mM NaCl, pH 7.0), and pure protein was identified using SDS-PAGE. Toyopearl HW-40C column was again used to buffer exchange EreB into storage buffer (20 mM HEPES, pH 7.5).

For EreC cells from 1 L of cell growth were resuspended in 30 mL of Ni NTA binding buffer (25 mM potassium phosphate, 150 mM NaCl, 30 mM imidazole pH 7.5). Cells were then lysed by sonication, and cellular debris was removed by centrifugation at 50,000 × *g* for 50 min at 4 °C. Residual cellular debris was removed via filtration through a 0.22 μm syringe-driven filter, and the resulting solution was applied on the XK 26/20 column containing 30 mL of Ni-NTA-Sepharose equilibrated in Ni NTA binding buffer. The protein was eluted using a buffer containing 25 mM potassium phosphate, 150 mM NaCl, 300 mM imidazole pH 7.5 in a step gradient. Fractions containing the protein of interest were identified using SDS-PAGE and pooled. EreC was subsequently buffer exchanged into ion exchange (IEX) buffer via dialysis in 10 L of IEX binding buffer (25 mM Bis-Tris, 50 mM NaCl, pH 7.22). The protein was again clarified using a 0.22μm syringe-driven filter and applied into an XK 26/40 column containing 100 mL of SP-Sepharose cation exchange resin. The protein was eluted using a gradient 0–100% elution buffer (25 mM Bis-Tris, 1050 mM NaCl, pH 7.22), and pure protein was identified using SDS-PAGE. The protein was subsequently exchanged into storage buffer by dialyzing pooled fractions into 12 L of 20 mM HEPES pH 7.0.

For EreD cells were lysed and clarified as described for EreC. Ni^2+^ affinity purification proceeded as previously mentioned for EreC. Fractions containing the protein of interest were identified using SDS-PAGE, pooled and concentrated. The protein-containing solution was clarified using centrifugation in a microcentrifuge at 12,000 × *g* for 15 minutes, and the clarified protein was applied on 26/60 Superdex 200 PG equilibrated with 20 mM HEPES pH 7.0. Purified protein was identified using SDS-PAGE, and pooled protein was stored in this form.

### Crystallization

EreC was crystallized at 22 °C using the sitting-drop diffusion method. The best quality crystal of EreC in crystal form I was obtained from a drop containing a 1:1 ratio of 10 mg mL^−1^ EreC H289N in storage buffer supplemented with 1 mM erythromycin, 2 mM tris(2-carboxyethyl) phosphine (TCEP) and reservoir solution consisting of 0.1 M phosphate-citrate pH 4.2, 5% (w/v) PEG 3000, 25% (v/v) 1,2-propanediol, 10% (v/v) glycerol.

The best quality crystal of EreC in crystal form II was obtained from a drop containing a 1:1 ratio of 10 mg mL^−1^ selenomethionine-labeled EreC H289N in storage buffer supplemented with 1 mM erythromycin, 2 mM tris(2-carboxyethyl) phosphine (TCEP), and reservoir solution consisting of 0.1 M sodium acetate pH 4.5, 5% (w/v) PEG 1000 and 50% (v/v) ethylene glycol. To obtain crystals of EreC in either crystal form it was essential to add erythromycin as an additive in the crystallization conditions.

### X-ray diffraction and structure refinement

The diffraction data for crystal form I EreC H289N was collected on a Bruker D8 Venture home source consisting of a METALJET X-ray source (liquid gallium anode) coupled with a PHOTON II detector. The dataset was integrated, scaled and reduced in the Bruker *PROTEUM3* suite (version 2018.7-2). The structure was solved by molecular replacement (MR) with the structure of distant homologue BcR135 (PDB ID: 3B55) as the search model using *PHASER* (version 2.8.3)^35^.

The diffraction data for crystal form II of selenomethionine-labeled EreC H289N were collected at the beamline 08B1-1 at the Canadian Macromolecular Crystallography Facility in Canadian Light Source synchrotron in Saskatoon, Canada using MxDC (version v2017.10-1334-g575fb36e). This crystal was grown with intention to solve the structure using single-wavelength anomalous diffraction; however, the first structure was solved in the meantime by using MR. The dataset was processed by using *xia2* (version 0.6.374) and *DIALS* (version 2.1.3) pipelines from the *CCP4* program suite (version 7.1)^36^. The crystal form II of EreC was solved by molecular replacement with crystal form I of EreC as the search model using *PHASER* (version 2.8.3)^35^.

Both structures were refined by iterative cycles of reciprocal-space refinement with *phenix.refine* (version 1.15.1-3469)^37^, and real-space refinement and model building in *Coot* (version 0.9)^38^. EreC crystal form I and crystal form II structure models were deposited in the PDB (PDB IDs: 6XCQ and 6XCS, respectively). The final data collection and refinement statistics are summarized in Table 2.

**Table 2:** Data collection and refinement statistics.

	Crystal form I	Crystal form II
	EreC^Closed^ H289N	EreC^Open^ H289N
	(PDB ID: 6XCQ)	(PDB ID: 6XCS)
Data collection statistics		
Resolution range (Å)	28.15–2.0 (2.072–2.0)	42.9–2.4 (2.486–2.4)
Space group	C 2 2 2_1_	C 1 2 1
Unit cell (Å, ˚)	68.4 92.7 125.8	95.3 74.2 129.2, β = 96.4
Total reflections	53938 (5398)	136190 (13203)
Unique reflections	27238 (2699)	35225 (3479)
Multiplicity	2.0 (2.0)	3.9 (3.8)
Completeness (%)	99.2 (99.9)	99.5 (98.5)
Mean I/sigma(I)	35.2 (8.6)	13.2 (1.3)
Wilson B-factor (Å^2^)	22.3	51.8
* R*-merge	0.032 (0.112)	0.101 (1.841)
* R*-meas	0.045 (0.158)	0.117 (2.142)
CC_1/2_	0.99 (0.98)	1.00 (0.68)
CC*	1.00 (1.00)	1.00 (0.90)
Refinement statistics		
* R*_work_	0.17 (0.21)	0.24 (0.40)
* R*_free_^a^	0.22 (0.27)	0.29 (0.49)
Number of non-hydrogen atoms	3585	6167
Macromolecules	3319	6103
Ligands	5	N/A
Water	261	64
Protein residues	408	816
RMS (bonds, (Å^2^))	0.007	0.008
RMS (angles, ˚)	0.89	1.23
Ramachandran favored (%)	95.1	96.7
Ramachandran outliers (%)	0.25	0.25
Clashscore	4.65	15.1
Average B-factor (Å^2^)	26.4	73.8
Macromolecules	25.7	73.9
Ligands	46.7	N/A
Solvent	34	66.3

Statistics for the highest-resolution shell are shown in parentheses.

^a^R_free_ was calculated by randomly omitting 10% of observed reflections from refinement.

### Molecular docking

The EreC in crystal form I was first mutated back into the WT variant of the protein using PyMOL, and all but one water hypothesized to be involved in catalysis were deleted from the structure. Erythromycin was prepared in UCSF Chimera (version 1.13.1) by adding hydrogens. The molecular surface of the protein was generated in UCSF Chimera, spheres were generated using the sphgen program, a grid box was generated, and the dimensions of the box set to encompass the entire active site of EreC. Molecular docking experiments were performed using DOCK (version 6.9). Flexible ligand docking calculations were performed, and a composite file containing all possible conformers were analysed in UCSF Chimera.

### Enzymatic activity assays

Liquid chromatography-mass spectrometry (LC-MS) was used to detect the inactivation products of macrolides by EreB, EreC, and EreD by using Agilent Technologies 1290 Infinity HPLC system coupled to Agilent Triple Quadripole mass spectrometer in positive ESI mode. Hundred micoliters of reaction mixture containing 2 mg mL^−1^ of Ere with 30 μM of antibiotic (dissolved in DMSO) were incubated for 60 minutes at room temperature in 20 mM HEPES, pH 7.5 buffer. The reaction was stopped by the addition of 300 µL of acetonitrile at −20 °C and clarified using centrifugation. 100 µL of the reaction was transferred into the tube, followed by 50 µL of water, resulting in a 1:1 (acetonitrile:water) ratio. Five microliters of the stopped reaction mix was loaded on a C18 column (ZORBAX RRHD Eclipse Plus C18 2.1 × 100 mm, 1.8 µm) and eluted using a gradient that employed the following two solutions: water with 0.05% (v/v) formic acid (A), and acetonitrile with 0.05% (v/v) formic acid (B). The details for the used gradient were: 5% solvent B from 0 to 2 min, a 2 to 10 min linear gradient up to 95% solvent B, 95% solvent B until 12 min, 5% solvent B from 12 to 16 min. For the duration of the experiment, the column was maintained at a temperature of 40 °C. The resulting data was obtained using Agilent Masshunter Workstation (version 10.0) and analyzed using Agilent MassHunter Quantitative Analysis (for QQQ) (version 10.0) to extract the area responsible for each peak corresponding to the substrate and the enzymatic product. Data ws further processed using Microsoft Excel (version 16.45). Experiments were performed in triplicate. Each macrolide was categorized either as a substrate, poor substrate, or non-substrate which was defined by their degradation rate, i.e.: >85% degraded, 30–85% degraded, and <30% degraded, respectively.

### Macrolide preparation for superimposition

In total, thirteen macrolides were used, where the coordinates of clarithromycin, azithromycin, spiramycin, josamycin, solithromycin, and telithromycin were available in the PDB. The structure of tulathromycin was generated by the addition of an *N*-methylpropylamine group onto the 5”-position of the cladinose sugar of azithromycin. The structure of roxithromycin was generated from erythromycin by replacing the oxygen atom bonded to C-9 of the macrolactone ring with nitrogen, to which, 2,4,7-trioxaoctane was attached. Oleandomycin, flurithromycin, and pikromycin were generated by modifying the scaffold of erythromycin and midecamycin from josamycin.

### Reporting summary

Further information on research design is available in the Nature Research Reporting Summary linked to this article.

## Supplementary information

Supplementary Information

Reporting Summary

## Data Availability

Coordinates and structure factors of EreC^Closed^ and EreC^Open^ have been deposited in the Protein Data Bank with accession codes 6XCQ and 6XCS, respectively. Sequences of EreA (UniProt P07684) EreB (UniProt P05789) EreC (UniProt C7C425) and EreD (UniProt A0A0H4JQQ7) were accessed using UniProt. Source data are provided with this paper. Additional data supporting the findings of this study are available from the corresponding author on reasonable request. Source data are provided with this paper.

## Source data

Source Data
