# Supplementary material for: Structural and functional insights into esterase-mediated macrolide resistance
Source: Nat Commun. 2021 Mar 19;12:1732. doi: 10.1038/s41467-021-22016-3 (PMC7979712; doi:10.1038/s41467-021-22016-3)
Supplement: Supplementary file 1 — Supplementary Information [file 41467_2021_22016_MOESM1_ESM.pdf]

Supplementary data

Structural and Functional Insights into Esterase-mediated Macrolide Resistance

Michał Zieliński[1,2], Jaeok Park†[1], Barry Sleno[1,2], Albert M. Berghuis*[1,2,3]

[1]Department of Biochemistry, McGill University, Montréal, Québec, Canada

[2]Centre de Recherche en Biologie Structurale, McGill University, Montréal, Québec, Canada

[3]Department of Microbiology and Immunology, McGill University, Montréal, Québec, Canada

*** Correspondence:**

Corresponding Author

albert.berghuis@mcgill.ca

† Present address: Department of Biochemistry, Memorial University of Newfoundland, St John's, Newfoundland and Labrador, Canada

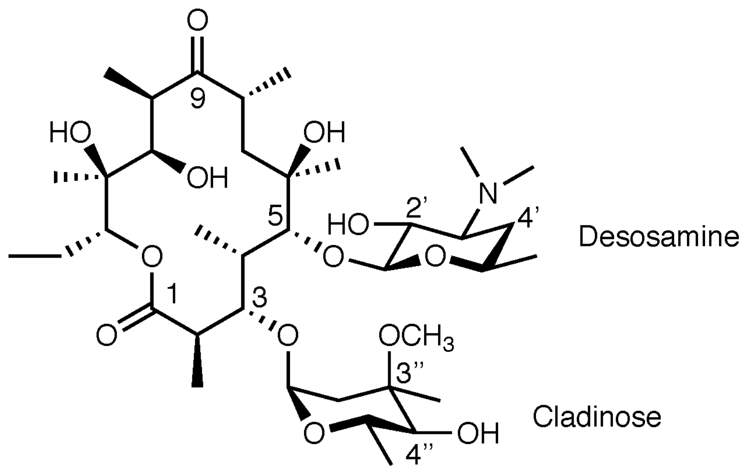

Supplementary figure 1: Carbon numbering of canonical macrolides, presented on erythromycin.

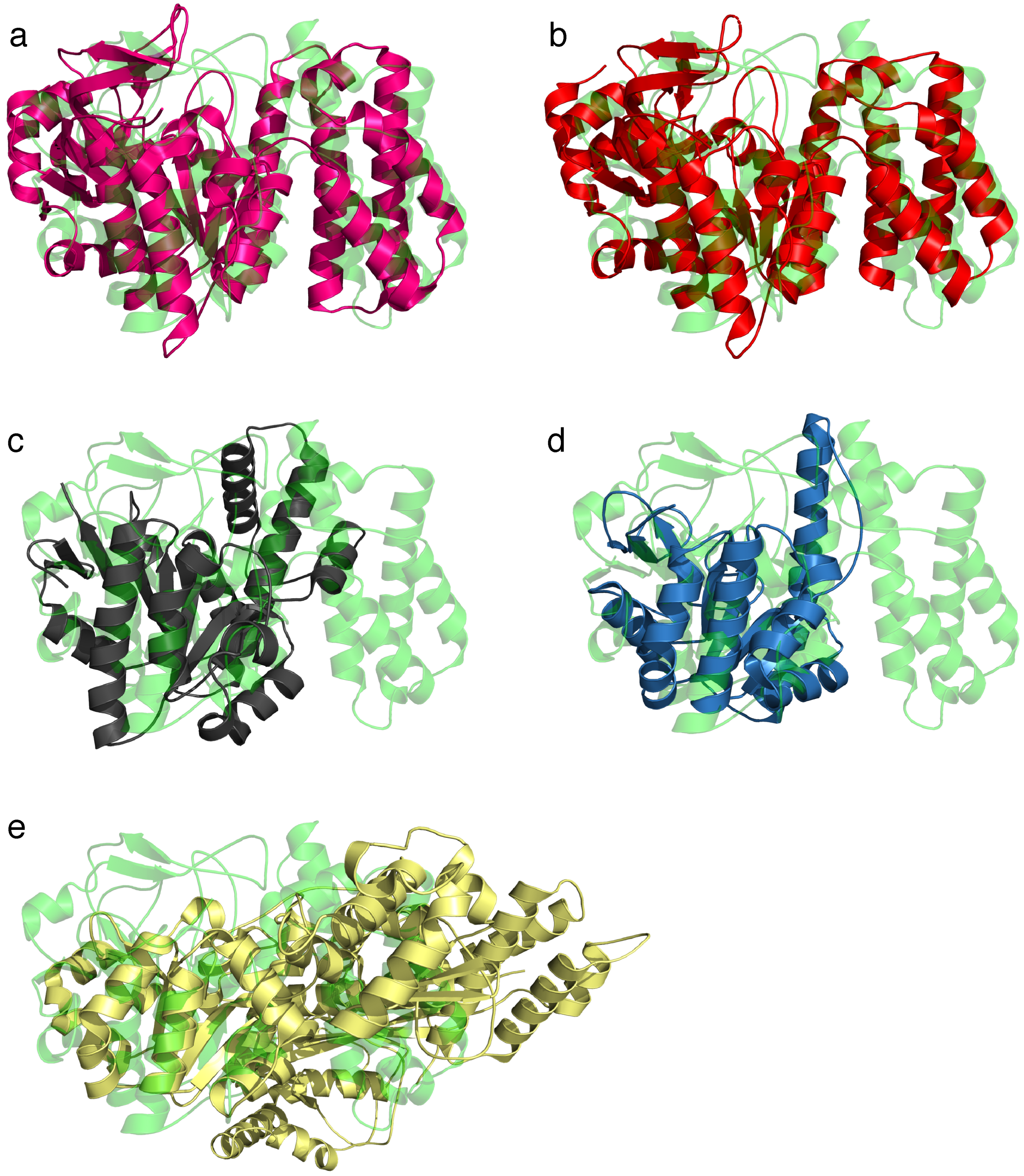

Supplementary figure 2: Comparison of folds displayed by distantly related proteins overlayed with EreC^{Closed} (green). A) BcR135 (PDB ID: 3B55) with RMSD 3.1 and DALI Z score of 35.7. B) BcR136 (PDB ID: 2QGM) with RMSD 3.2 and DALI Z score of 36.0. C) ChaN (PDB ID: 2G5G) with RMSD 3.5 and DALI Z score of 13.6. D) HopBA1 (PDB

ID: 5T09) with RMSD 3.5 and DALI Z score of 11.3. E) PMT (PDB ID: 2EBF) with RMSD 4.3 and DALI Z score of 9.6.

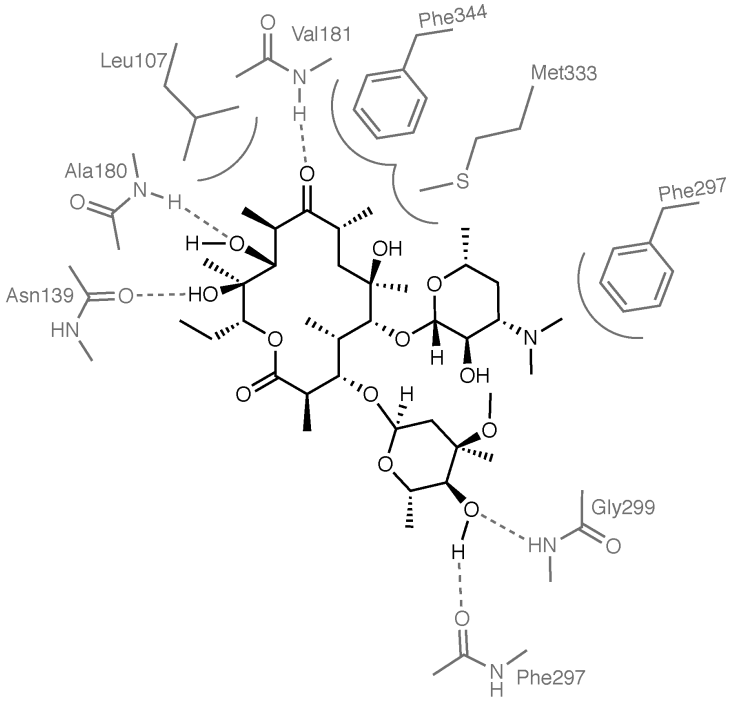

Supplementary figure 3: Non-catalytic interactions modelled between EreC and erythromycin using Dock6.

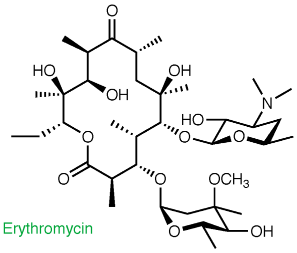

Erythromycin

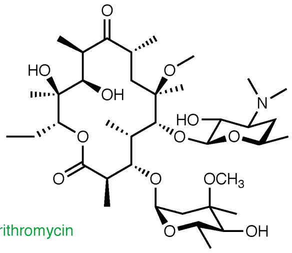

Clarithromycin

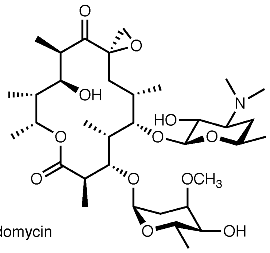

Oleandomycin

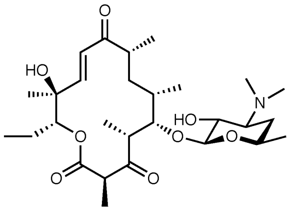

Pikromycin

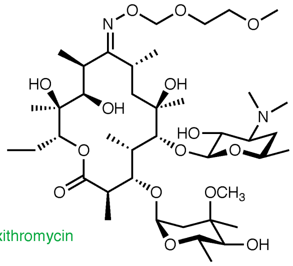

Roxithromycin

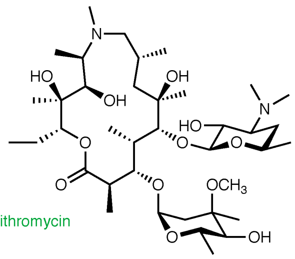

Azithromycin

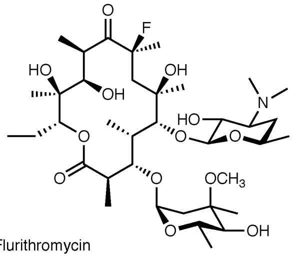

Flurithromycin

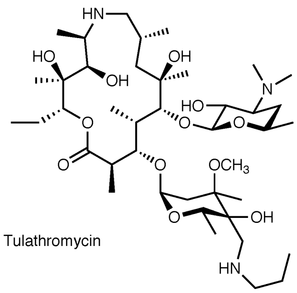

Tulathromycin

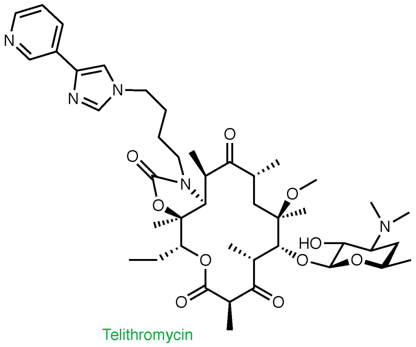

Telithromycin

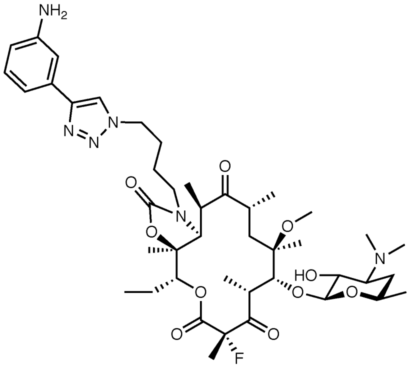

Solithromycin

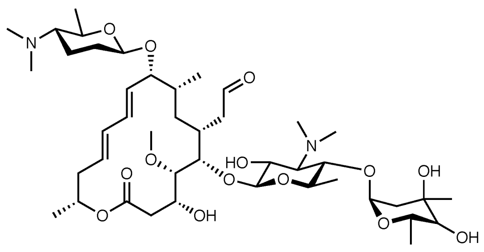

Spiramycin I

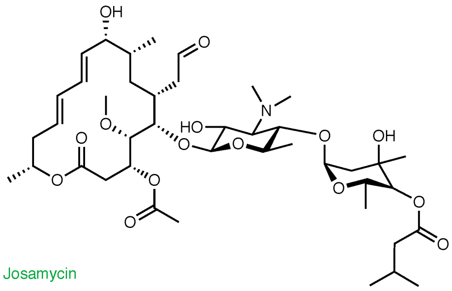

Josamycin

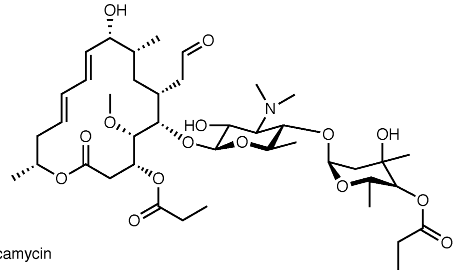

Midecamycin

Supplementary figure 4**. Macrolides used in this study.** A panel of thirteen macrolides used in this study, clinically relevant macrolides are identified in green.

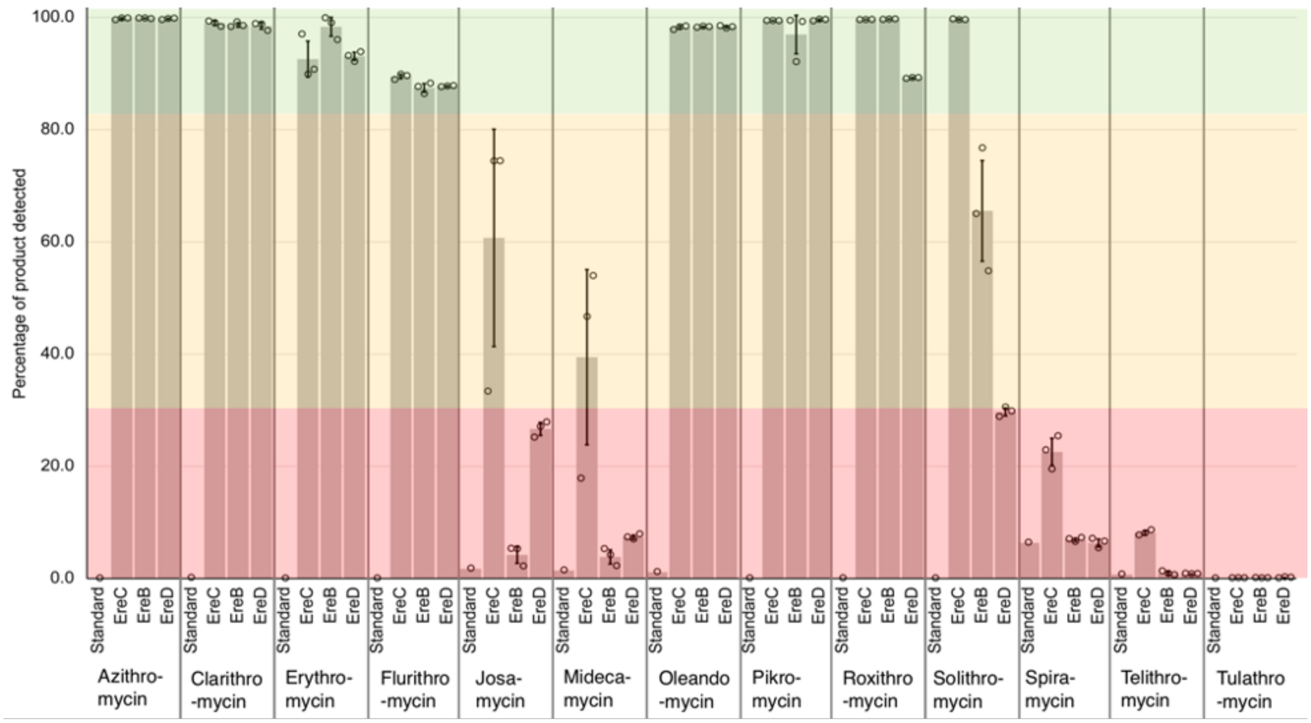

Supplementary figure 5: Percentage of product detected using LC-MS, where thirteen macrolides were subjected to action of EreB, EreC and EreD. Substrates are classified as substrate (green) if >85% of macrolide was degraded, poor substrate (orange) for 30-85% degraded and non-substrate (red) for <30% degraded. Enzyme with macrolide n=3, macrolide standard n=1. Error bars represent standard deviation. Centre of the error bar is represented by the average of the data points. Source data are provided as a Source Data file.

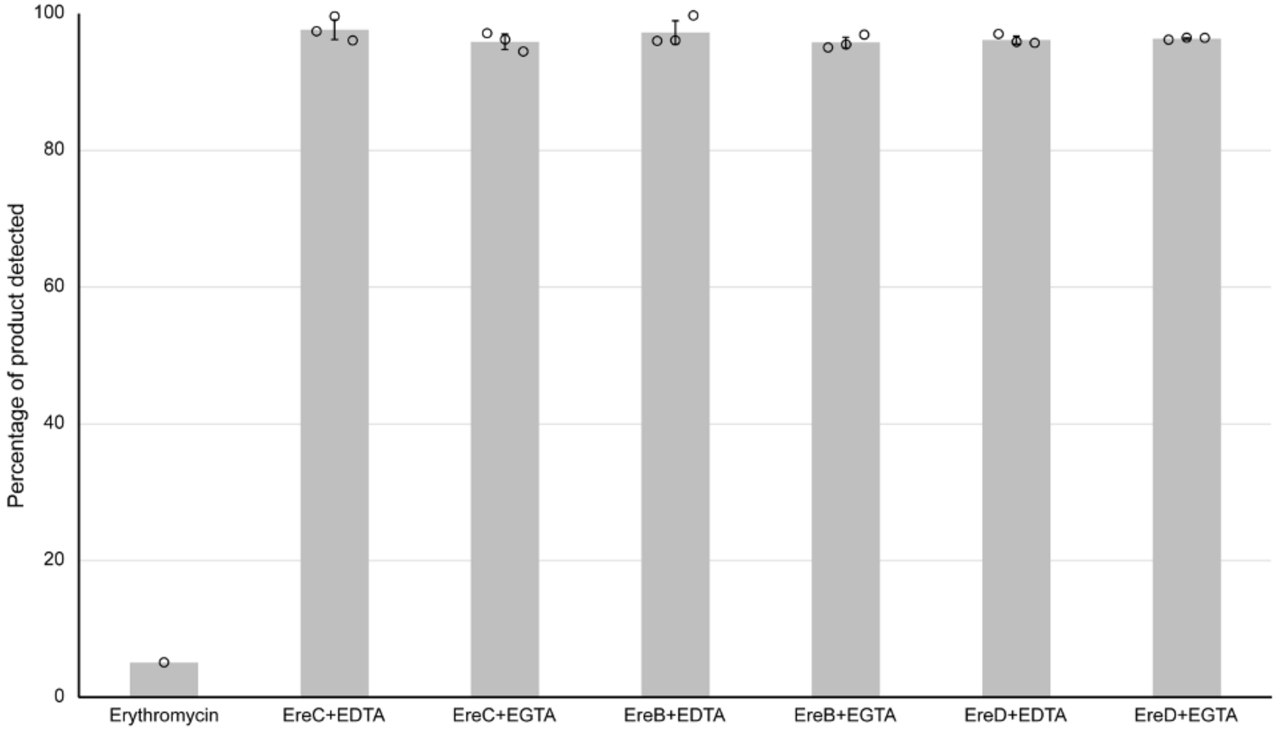

Supplementary figure 6 Percentage of product detected using LC-MS, where erythromycin was subjected to action of EreB, EreC and EreD in presence of chelating agents; EDTA and EGTA. Enzyme with macrolide n=3, macrolide standard n=1. Error bars represent standard deviation. For metal dependency experiments, 2mM EDTA or EGTA were incubated with enzymes for one hour before adding erythromycin, followed by the standard protocol for the addition and detection of product/substrate, as shown in enzymatic activity assays section of methods. Centre of the error bar is represented by the average of the data points. Source data are provided as a Source Data file.
